# Cancer stem cell markers are enriched in normal tissue adjacent to triple negative breast cancer and inversely correlated with DNA repair deficiency

**DOI:** 10.1186/bcr3471

**Published:** 2013-09-04

**Authors:** Rachel L Atkinson, Wei T Yang, Daniel G Rosen, Melissa D Landis, Helen Wong, Michael T Lewis, Chad J Creighton, Krystal R Sexton, Sue G Hilsenbeck, Aysegul A Sahin, Abenaa M Brewster, Wendy A Woodward, Jenny C Chang

**Affiliations:** 1 Departments of Clinical Cancer Prevention, The University of Texas MD Anderson Cancer Center, Houston, TX, USA; 2 Departments of Diagnostic Imaging, The University of Texas MD Anderson Cancer Center, Houston, TX, USA; 3 Departments of Radiation Oncology, The University of Texas MD Anderson Cancer Center, Houston, TX, USA; 4 Departments of Pathology, The University of Texas MD Anderson Cancer Center, Houston, TX, USA; 5 Lester & Sue Smith Breast Center, Baylor College of Medicine, Houston, USA; 6 Pathology, Baylor College of Medicine, Houston, TX, USA; 7 The Methodist Hospital Research Institute, 6445 Main Street, 21st Floor, Houston, TX 77030, USA

## Abstract

**Introduction:**

We hypothesized that cells present in normal tissue that bear cancer stem cell markers may represent a cancer cell of origin or a microenvironment primed for tumor development, and that their presence may correlate with the clinically defined subtypes of breast cancer that show increased tumorigenicity and stem cell features.

**Methods:**

Normal tissues sampled at least 5 cm from primary tumors (normal adjacent tissue) were obtained from 61 chemotherapy-naive patients with breast cancer treated with mastectomy. Samples were stained simultaneously with immunofluorescence for CD44/CD49f/CD133/2 stem cell markers. We assessed the association between CD44^+^CD49f^+^CD133/2^+^ staining in normal adjacent tissue and breast cancer receptor subtype (defined by the expression of the estrogen (ER), progesterone (PR), or human epidermal growth factor-2 (Her2) receptors). We also examined the correlation between CD44^+^CD49f^+^CD133/2^+^ immunofluorescence and each of two previously published gene signatures, one derived from stem-cell enriched tissue and one from BRCA mutated tissue expected to have defective DNA repair.

**Results:**

Patients with triple negative breast cancer (ER^–^/PR^–^/HER2^–^) expressed CD44^+^CD49f^+^CD133/2^+^ in 9 of 9 normal adjacent tissue samples compared with 7 of 52 ER^+^ and/or Her2^+^ tumors (*P* < 0.001). Further, expression of CD44^+^CD49f^+^CD133/2^+^ by normal adjacent tissue correlated positively with a stem cell-derived tumorigenic signature (*P* <0.001) and inversely with a defective DNA-repair signature (*P* <0.001).

**Conclusion:**

Normal cells bearing cancer stem cell markers are associated with the triple negative receptor subtype of breast cancer. This study suggests stem cell staining and gene expression signatures from normal breast tissues represent novel tissue-based risk biomarkers for triple negative breast cancer. Validation of these results in additional studies of normal tissue from cancer-free women could lay the foundation for future targeted triple negative breast cancer prevention strategies.

## Introduction

Breast cancer is a heterogeneous disease consisting of various receptor subtypes that have different treatment options and long-term survival probabilities. Clinically defined subtypes have been described on the basis of immunohistochemical expression of the estrogen receptor (ER), progesterone receptor (PR), and human epidermal growth factor receptor-2 (Her2). The so-called triple-negative subtype (ER^–^/PR^–^/Her2^–^) accounts for fewer than 20% of breast cancer cases. However, because of its poor prognosis and lack of treatment options triple-negative disease is responsible for a disproportionate number of breast cancer deaths [1]. Although chemoprevention strategies with tamoxifen and exemestane are available to suppress or prevent the development of ER-positive tumors, no chemoprevention measures are available for triple-negative breast cancer [2].

Increasing evidence from recent clinical studies supports the hypothesis that breast tumors contain a subpopulation of cells with distinct properties similar to stem cells (reviewed in [3]). The various combinations of surface markers used to identify breast cancer stem cells include CD44^+^/CD24^-/low^, aldehyde dehydrogenase (ALDH1) activity (identified by the Aldefluor assay), and more recently CD44^+^/CD49f^+^/CD133/2^+^; all define subpopulations of cells that have been shown in limiting dilution xenograft transplantation assays to have increased tumorigenic potential [4-7]. Differences in results obtained from experimental studies to date suggest that these markers may differ depending on tumor type.

We previously generated a breast cancer stem-cell signature that comprised 493 differentially expressed genes, patterns of which were present in a tumorigenic subpopulation of cells identified by using putative stem-cell markers [8]. The breast cancer stem cell signature was generated by combining two subpopulations of 36 breast tumors sorted for CD44^+^/CD24^-/low^ markers and the cells that were able to form mammospheres on low-attachment plates. The tumors collected represented all subtypes of breast cancer (18 luminal A/B, 13 basal-like, and 5 Her2 cancers). Comparative gene expression profiling was performed on these populations and compared to the bulk of the tumor (non-CD44^+^/CD24^-/low^ cells) and analyzed for significant overlap between the gene expression patterns. When compared with breast tumors, this signature was found to correlate most closely with gene expression patterns in tumors identified as claudin-low molecular subtype. Functionally, stem cells are associated with enhanced DNA repair, which may explain their resistance to both chemotherapy and radiation [9,10]. We previously identified a defective DNA damage repair signature based on published *BRCA* signatures with a subset of the 69 most differentially expressed genes [11]. This signature represents a BRCA-like phenotype expected to be associated with defective DNA damage repair; clinically, it has been associated with poor response to anthracyclines [11].

Triple-negative breast cancer and the epidemiologic factors associated with it are different from those of other breast cancer subtypes [12-14]. Millikan *et al*. observed in the Carolina Breast Cancer Study that African American ethnicity, higher numbers of pregnancies, younger age at full-term pregnancy, and shorter duration of breastfeeding were associated with a higher risk of triple-negative breast cancer [14]. A more recent study by Rosenberg’s’ group found a similar association with reproductive risk factors in a cohort of African American women [15]. Full-term pregnancy followed by little or no breastfeeding has been proposed to lead to the retention of the normal stem cell pool intended to transiently expand to support lactation and which is speculated to contract with extended breastfeeding. This could preferentially lead to the development of triple-negative breast cancer, which is most closely associated with a cancer stem-cell phenotype [16-20].

The idea that some breast cancers develop from stem cells is controversial and highly dependent on the subtype of breast cancer. In this study, we investigated normal tissue samples adjacent to breast tumors in women with a variety of subtypes of breast cancer. We hypothesized that the adjacent normal breast tissue in women with triple-negative breast cancer may contain increased numbers of cells bearing cancer stem-cell markers.

## Methods

### Patient samples

We randomly identified 61 patient samples from a tissue bank of normal breast tissue obtained from mastectomy specimens from women undergoing surgery for breast cancer from July 2005 through July 2007. These biopsy specimens had been obtained at least 5 cm away from the tumor. Tissue samples from 11 women who underwent prophylactic contralateral mastectomy were also analyzed. The normal adjacent breast tissue had been collected in optimum cutting temperature cryoembedding medium and formalin and then embedded in paraffin. A section of each specimen was verified by two independent pathologists, who used hematoxylin and eosin staining according to standard protocols. Only samples containing normal breast tissue with at least 10 to 20% epithelial content, with associated 60% tumor cellularity were selected and used for both staining and assessment of gene expression. The investigators were blinded to patient identity and the clinicopathologic features of the tumor for imaging and analysis. This study was approved by the appropriate institutional review board of The University of Texas MD Cancer Center. All patients gave their written informed consent prior to study inclusion.

### Gene expression

The methods for gene expression experiments have been described previously [21]. Briefly, total RNA was isolated using TRIzol (Invitrogen Corp., Carlsbad, CA, USA). Samples were then passed over Qiagen RNeasy (Qiagen, Valencia, CA, USA). After RNA recovery with a T7 promoter primer, cDNA was synthesized and fluorescently labeled, and the labeled cRNA samples were hybridized onto an (Affymetrix, Santa Clara, CA) U133 Plus2 GeneChip, which contains approximately 34,000 genes, according to the manufacturer’s recommended procedures. The median amount of RNA extracted was 25 μg (range 19 to 33 μg). The quality of the RNA was good, as previously published, the percent call was high at between 40 and 50%, and the glyceraldehyde-3-phosphate dehydrogenase (GADPH) ratio was below 1.0. dChip was used to estimate expression values by using the perfect match/mismatch (PM/MM) difference model and invariant set normalization [21].

### Immunohistochemical analysis

Approximately six different areas of epithelial cells from each sample were scored, with each area consisting of approximately 200 cells. Immunofluorescence staining for CD44, CD49f, CD133/2, and 53BP1 was performed according to previously published methods [7,22] with the following antibodies: rabbit anti-CD44 (Abcam, Cambridge, MA), rat anti-CD49f (BD Biosciences, San Jose, California), mouse anti-CD133/2 (Miltenyi Biotech, Auburn, CA), and rabbit anti-53BP1 rabbit polyclonal (Novus Biologicals, Littleton, CO). All secondary antibodies were purchased from (Invitrogen, Grand Island, NY): anti-rabbit Alexa Fluor 594, anti-rat Alexa Fluor 647, or anti-mouse Alexa Fluor 488. A positive score was defined as more than 1% positive staining and a negative score was assigned for less than 1% positive cells.

### Microscopy

Microscopic analysis was performed at 40× magnification with a Leica TCS SP5 confocal microscope and a Leica LAS AF to detect the expression of CD44, CD49f, and CD133/2. Microscopic analysis was also done at 40× magnification with an Olympus IX-81 DSU confocal microscope using 3I’s (Slidebook Software (V5.0), Denver, CO) to quantify 53BP1.

### Statistical methods

Frequency counts and summary statistics were calculated for all clinical and epidemiologic data, and the variables were compared between stem cell-positive and stem cell-negative tissue samples. Associations between the tumorigenic signature and stem cell staining were assessed by using the Wilcoxon rank sum test. Given a previously defined gene expression signature of interest (for example, for tumorigenic cells or DNA damage response), we scored each of the normal adjacent breast tissue expression profiles for similarity to the signature pattern by using our previously described *t*-score metric [8,23]. Briefly, the gene signature *t*-score was defined as the two-sided *t*-statistic comparing the average of the signature-high genes with that of the signature-low genes within each tumor (after first centering the log-transformed expression values for the breast dataset on the median across samples).

As exploratory analyses, Kaplan-Meier curves were generated for the stem cell-positive and stem cell-negative groups, and the log-rank test was used to determine significant differences in survival times between the two groups. Recurrence-free survival time was defined as the time from the date of the initial diagnosis of breast cancer to the date of distant or local recurrence, whichever came first. Subjects who were alive and recurrence-free at the last follow up were censored on that date. All *P*-values were two-sided with *α* = 0.05, and all statistical analyses were conducted using STATA 10.0 (College Station, TX, USA). Graphs were generated using GraphPad Prism version 4.00 for Macintosh (GraphPad Software, San Diego, CA, USA; http://www.graphpad.com).

## Results

### Increased prevalence of cancer stem-cell markers in the normal adjacent tissue of patients with triple-negative breast cancer

**Figure 1:**
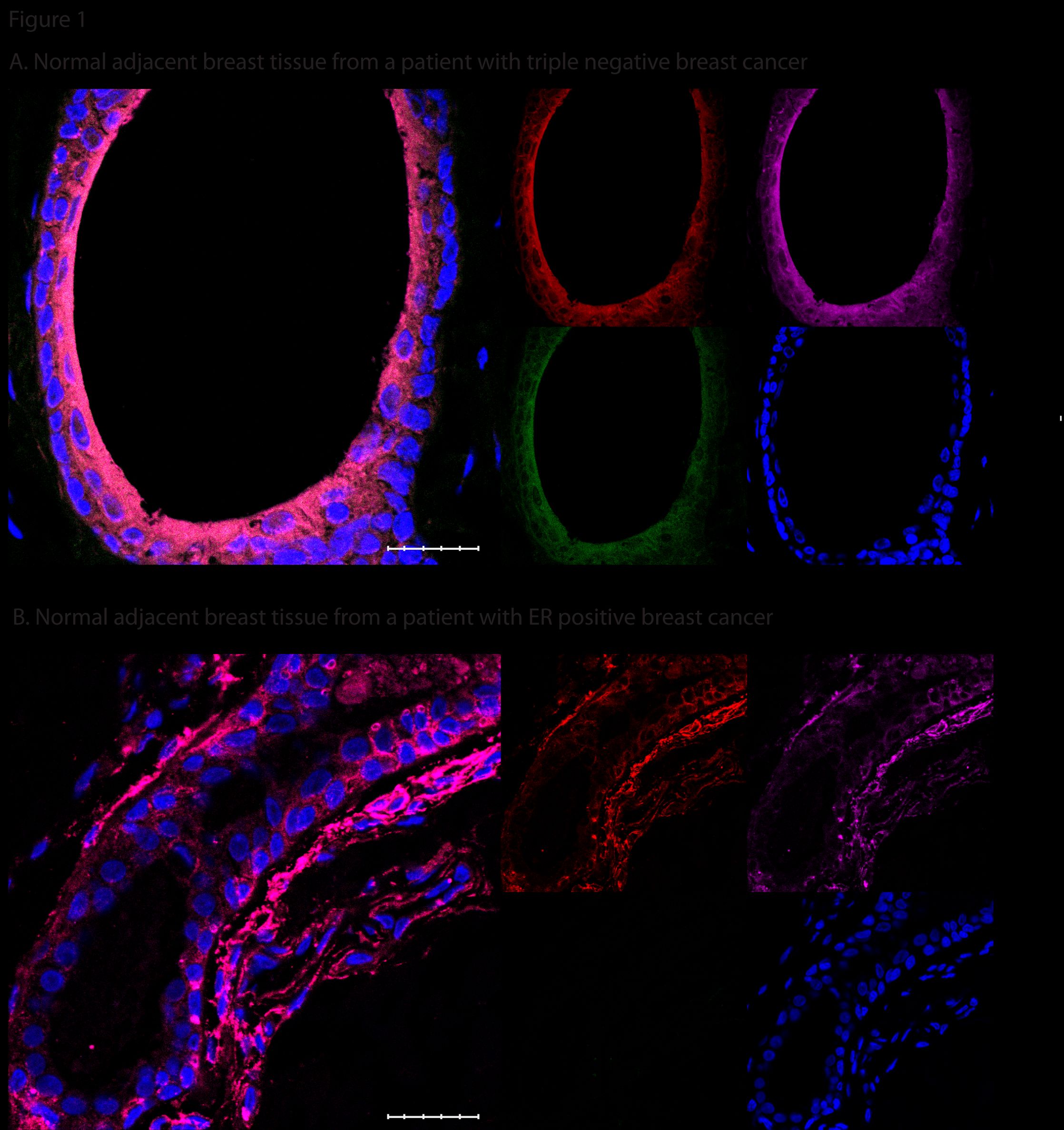
**Stem cell cell surface markers were present in the triple negative normal adjacent tissues to a much greater extent than in the estrogen receptor-positive (ER**^**+**^**) sample. (A)** Normal adjacent tissue from a patient with triple-negative cancer: red, CD44; purple, CD49f; green, CD133/2; blue, 4',6-diamidino-2-phenylindole (DAPI) and combined CD44^+^/CD49f^+^/CD133/2^+^. Scale bar 25 μm (40×). **(B)** Normal adjacent tissue from a patient with ER^+^ disease, no combined cells CD44^+^/CD49f^+^/CD133/2^+^. Scale bar 25 μm (40×).

The presence of the cell surface markers CD44^+^/CD49f^+^/CD133/2^+^ was significantly increased in normal adjacent tissue associated with patients with triple-negative breast cancer (nine of nine) compared with that in ER^+^ and Her2^+^ tumors combined (seven of fifty-two) (*P* <0.001); representative samples are shown in Figure 1 and Additional file 1. The average number of fields positive in normal adjacent tissue with at least 1% positive staining was 60.5% (range 20.0 to 100.0%). The average number (range) of cells positive for CD44, CD49f, and CD133 in normal adjacent tissue associated with triple-negative cancer was 66.0% (20.0 to 100.0%), 57.0% (20.0 to 80.0%), and 52.0% (10.0 to 80.0%), with no significant differences between the expression of the markers. In pairwise comparisons between the tissues positive for CD44^+^/CD49f^+^/CD133/2^+^ there was no significant difference in the expression of individual markers between the triple-negative and ER^+^ tissues. Staining prevalence and patterns in all cases where a prophylactic contralateral mastectomy sample was available were similar to the staining on the paired ipsilateral side, including the two samples from patients with triple-negative disease (data not shown).

### Tumorigenic signature in normal adjacent breast tissue with positive staining for CD44^+^/CD49f^+^/CD133/2^+^

**Figure 2:**
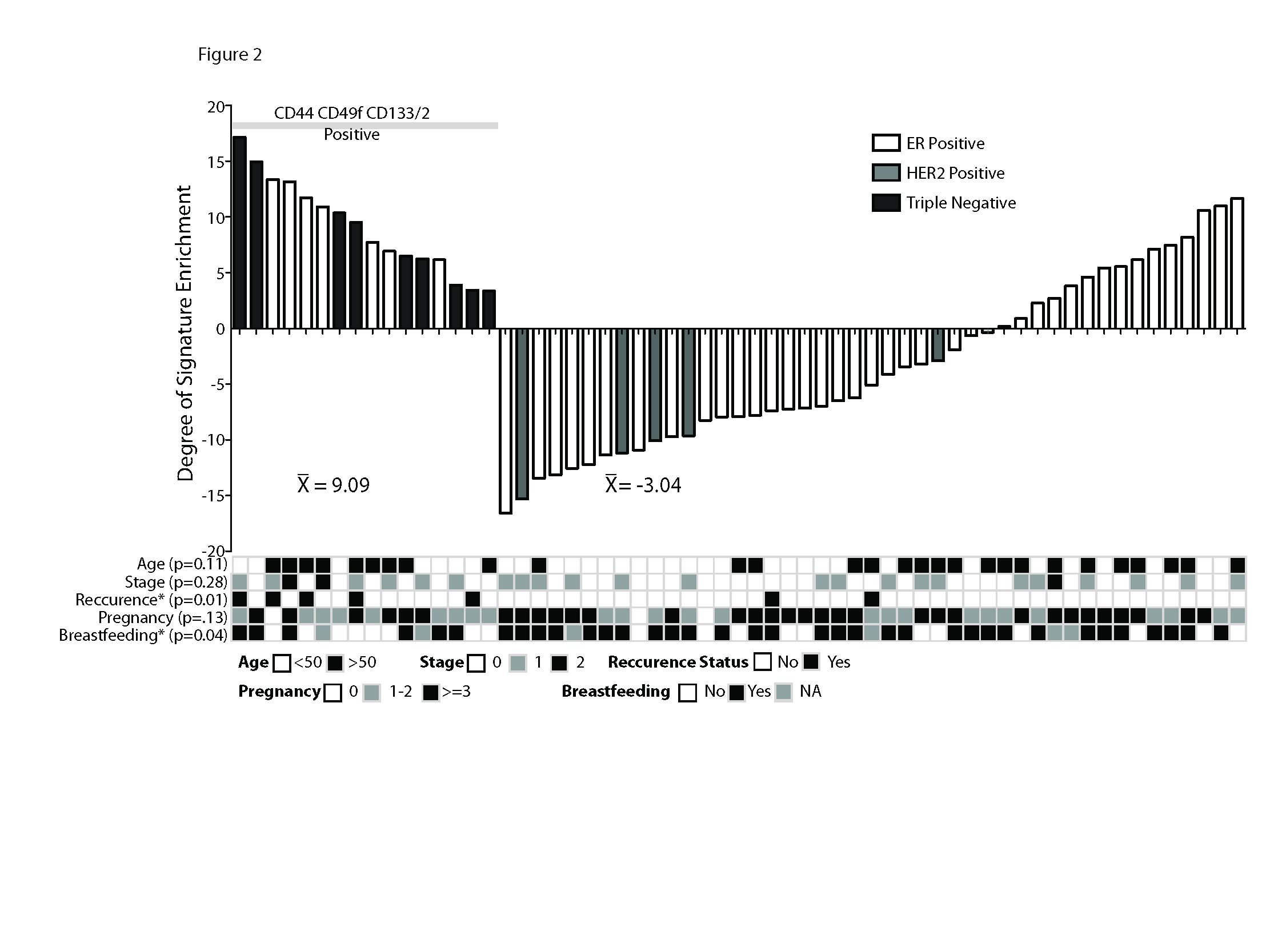
**Gene expression patterns associated with tumorigenic cells are manifested in normal breast samples with positive staining for the stem-cell markers CD44**^**+**^**/CD49f**^**+**^**/CD133/2**^**+**^**.** On the basis of gene expression profiling, normal breast samples were each assigned a score for similarity to a previously defined gene signature of tumorigenic cells. CD44^+^CD49f^+^CD133/2^+^ samples scored highly for the gene signature (*P* <0.001, Wilcoxon rank sum test): black bar, normal adjacent tissue. Analysis of epidemiologic factors revealed associations between stem cell presence and triple-negative tumor subtype (*P* = 0.001), breastfeeding (*P* = 0.04) and recurrence (*P* = 0.01). ^*^P <0.05.

We scored the gene expression profiles of the normal adjacent breast specimens for expression of a tumorigenic stem-cell signature, and found a strong correlation between high signature manifestation and CD44^+^CD49f^+^CD133/2^+^ stem-cell staining (Figure 2, *P* <0.001). The nine normal adjacent breast specimens from patients with triple-negative breast cancer, which were all positive for CD44^+^CD49f^+^CD133/2^+^ stem cell staining, also all exhibited high tumorigenic signature patterns. In addition, all seven of the normal adjacent tissues from women with ER^+^ breast cancer that were positive for CD44^+^CD49f^+^CD133/2^+^ also exhibited high tumorigenic signature patterns. These data support the concept that the tissues identified by immunofluorescence as positive for stem-cell markers also exhibit a stem cell-like gene expression profile. In normal adjacent tissue from women with ER^+^ breast cancer, 15 out of 45 were positive for the stem cell-like gene expression profile without expression of CD44^+^CD49f^+^CD133/2^+^. The average tumorigenic signature score for tissues stained positive for CD44^+^CD49f^+^CD133/2^+^ was 9.09 and the average for tissues stained negative was -3.04 (Figure 2, *P* <0.001, Wilcoxon rank sum test).

### DNA damage and function

**Figure 3:**
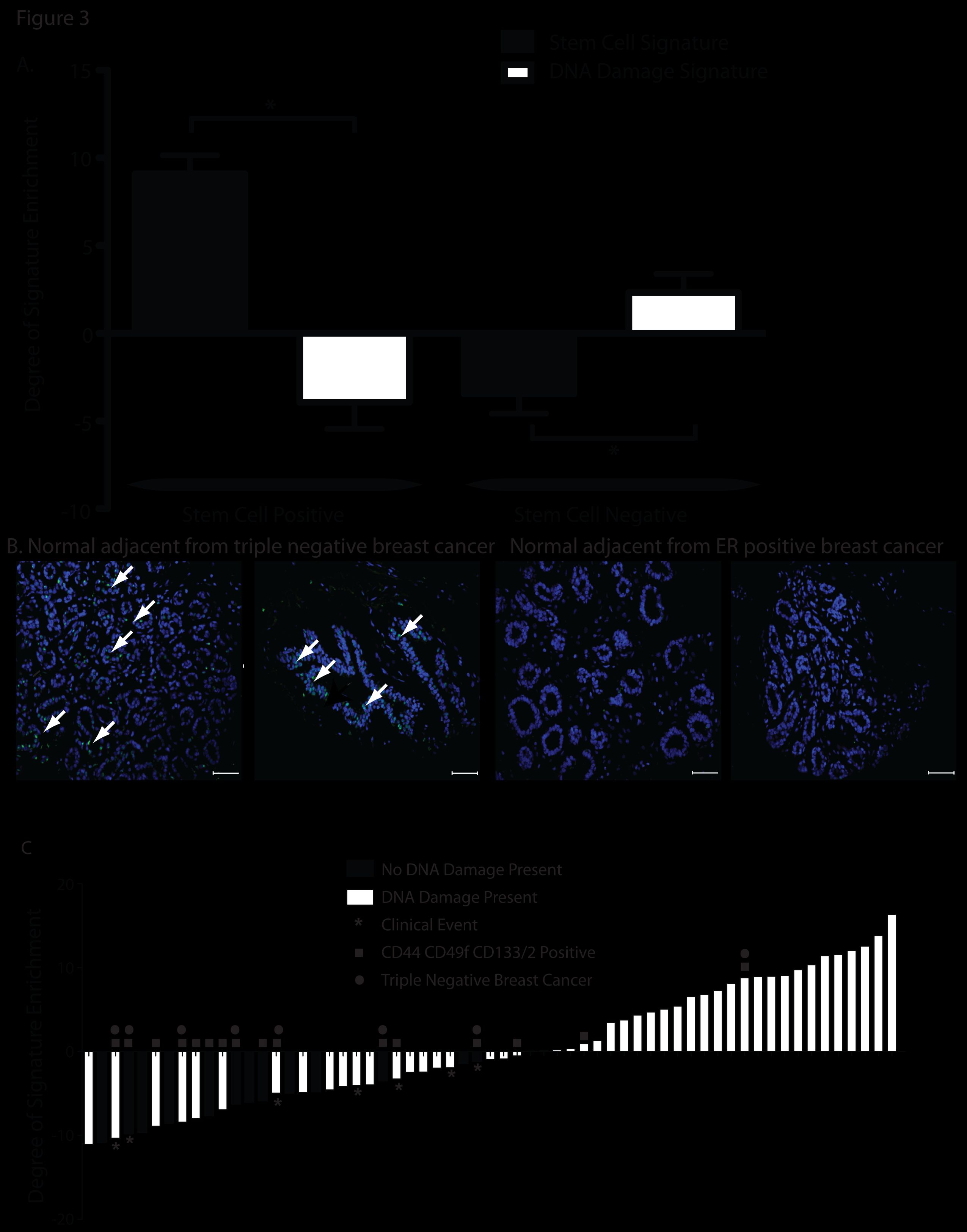
**Lack of DNA damage found in stem cell-positive tissues. (A)** Inverse correlation between stem-cell signature (black bar) and defective DNA-repair signature (white bar); ^*^P = 0.001. **(B)** 53BP1 staining in normal adjacent tissue. Green foci (arrows) indicate DNA damage, scale bar 50 μm. **(C)** The presence of 53BP1 foci correlates significantly with the defective DNA repair signature (*P* <0.001, Wilcoxon rank sum test). White bars, DNA damage present; black bars, no DNA damage present; asterisks indicate a recurrence event (all events were within the cohort with negative scores); closed circles indicate triple-negative breast cancer; closed squares indicate CD44^+^/CD49f^+^/CD133/2^+^.

Tissues characterized for stem-cell marker staining and gene expression were also characterized for the presence of DNA damage as assessed by 53BP1 immunohistochemical staining and the expression of a defective DNA repair signature. Within the normal adjacent breast specimens, the defective DNA damage signature patterns were inversely related to the stem-cell signature patterns (Figure 3A, *P* <0.001). All of the tissues were stained with 53BP1 to validate the DNA damage signature with a protein-based repair surrogate (Figure 3B). Significantly positive defective DNA repair-gene expression was associated with an abundance of DNA damage (53BP1 foci) (Figure 3C). Interestingly, all of the clinical events (local recurrence, distant metastasis, and death from cancer) took place among women whose samples had a negative defective DNA damage score.

### Epidemiologic factors and survival analysis

**Figure 4:**
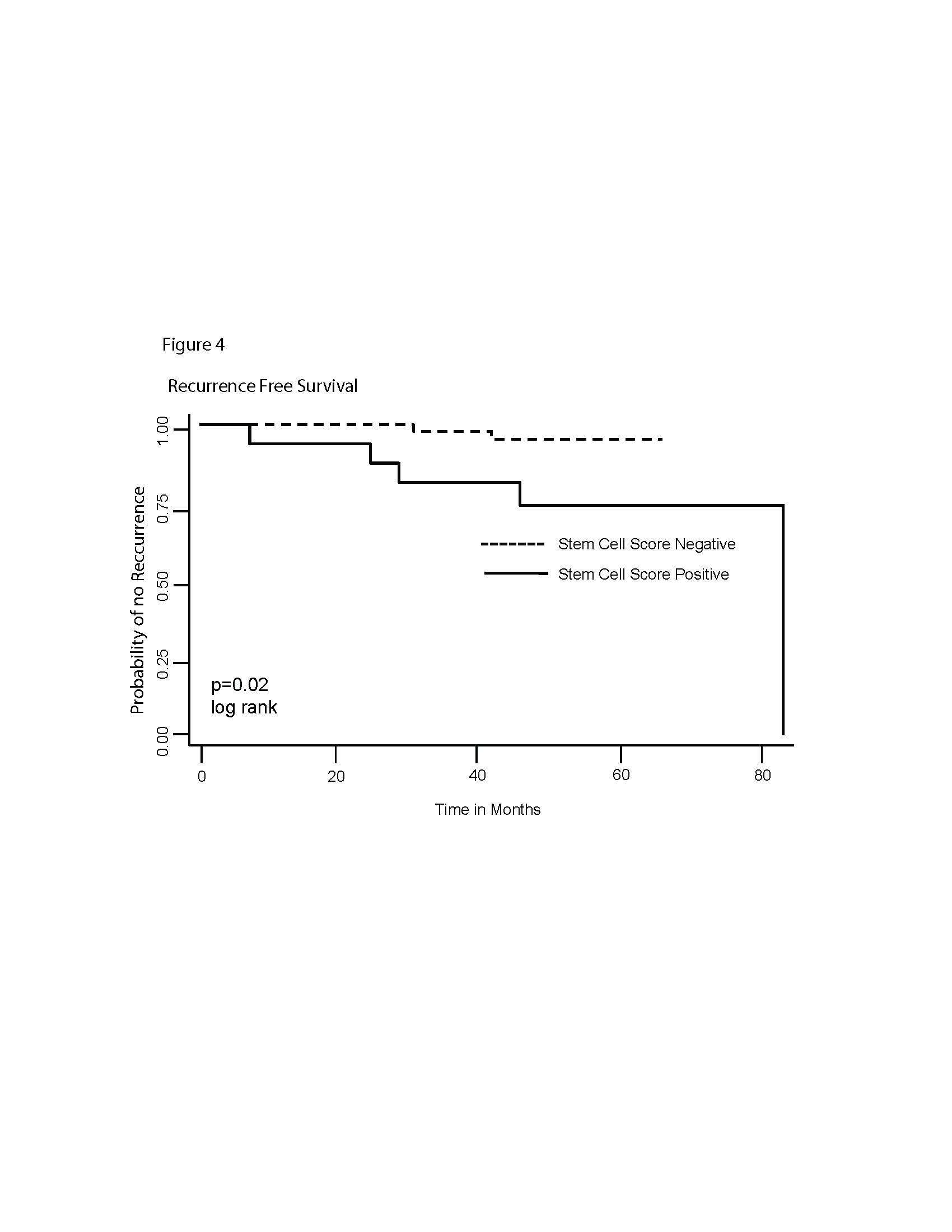
**Recurrence-free survival among all patients, grouped by stem cell-staining status.** Dashed lines, stem cell-negative group; solid lines, stem cell-positive group. Recurrence-free survival was better among patients with cells staining positive for stem-cell markers (*P* = 0.02).

Comparison of epidemiologic factors between patients with stem-cell marker-positive or stem-cell marker-negative normal adjacent tissue revealed that marker-positive tissue was associated with never breastfeeding (*P* = 0.04) and shorter breastfeeding duration per child (1.8 ± 2.9, 4.5 ± 6.1 months, *P* = 0.05) in the stem-cell marker-positive cohort (Figure 2). Although this study was not powered to compare recurrence-free survival between subgroups, the outcomes did seem to be different for patients with tissue that stained positively versus negatively for the presence of stem cells (Figure 4) (*P* = 0.02). The risk of recurrence was higher in those with stem cells present (hazard ratio = 6.11, 95% CI 1.12, 33.37), but there was no association between triple-negative disease and breast cancer recurrence.

## Discussion

We found that the normal adjacent tissue associated with triple-negative breast cancer contains cells that express the same cell-surface markers as do triple-negative breast cancer stem cells and also shows global gene expression that closely correlates with a previously published tumorigenic cancer stem-cell signature. In addition, both a defective DNA-repair gene expression signature as well as 53BP1 staining indicated that normal adjacent tissue associated with triple-negative breast cancer had enhanced DNA repair compared with normal adjacent tissue from women with ER^+^ or Her2^+^ breast cancer. Although correlative, these findings are consistent with the hypothesis that stem cells present in normal breast tissue that is pathologically without evidence of any tumor cells may be either the cell of origin for breast cancer or promote a tumor-conducive microenvironment necessary for the development of breast cancer.

Interestingly, the risk factors associated with the development of triple-negative breast cancer are different from those for breast cancer as a whole [24]. Several epidemiologic studies have shown that women with higher numbers of pregnancies, younger age at full-term pregnancy, and shorter duration of breastfeeding are at increased risk for triple-negative breast cancer [14,15]. During pregnancy, progenitor cells in the breast terminal ducts proliferate and undergo terminal differentiation, apoptosis, or both, during lactation and involution [16]. Although the biological mechanism underlying this process is not well-understood, investigations with various mouse models have demonstrated that pregnancy and lactation affect the number and differentiation status of breast stem cells [25-27]. In our study, both the staining for stem cells and the associated gene signature were disproportionately higher in normal adjacent breast epithelium associated with triple-negative breast cancer. Our results are consistent with our hypothesis that breast epithelium from patients with triple-negative disease contains more stem cells and that their presence may be linked to reproductive factors. Consistent with this hypothesis, we found that patients with stem cells in the normal adjacent tissue were less likely to have breastfed and had a shorter duration of breastfeeding. These results raise interesting questions regarding the prevention of triple-negative breast cancer. Future studies will attempt to identify these cells in biopsies from patients without breast cancer and to correlate these to the development of breast cancer in high-risk cohorts.

The results from immunofluorescence staining for stem-cell markers strongly support the hypothesis that women with triple-negative breast cancer have more stem cells present in their normal breast tissue than do women with other types of cancer. Although the normal epithelial hierarchy is a useful framework to understand the cellular origins of the different subtypes of breast cancer, these findings remain correlative until formally proven by assessing the tumorigenic capability of these different cell populations or their assessment in patients without tumors. Helland *et al*. recently investigated gene expression patterns in normal breast biopsy samples [28] and identified a subset of women with tissues enriched with a claudin-low signature. An alternative explanation for our findings is that the presence of stem-cell markers as well as the tumorigenic signature in the normal adjacent tissue reflect the tumor’s influence on the adjacent normal tissue. However, the similar staining patterns and gene expression signatures in the contralateral normal breasts of women who underwent contralateral prophylactic mastectomy make this less likely. If indeed the former is true then this also suggest the location of the tumor may be random, given the wide spatial dispersal of stem cells found within the normal breast tissue.

The results on the DNA-damage signature are consistent with the stem cell hypothesis and have an interesting bearing on ongoing studies of poly (adenosine diphosphate-ribose) polymerase (PARP) inhibitors in triple-negative breast cancer. An initial phase II clinical trial using iniparib, a PARP inhibitor, with gemcitabine showed clinical benefit and improvement in survival for women with metastatic triple-negative breast cancer, but further studies have not confirmed that PARP inhibitors provide a progression-free survival benefit in triple-negative breast cancer [29]. The mixed results from clinical trials suggest that a specific subpopulation of patients may benefit from the PARP inhibitors, perhaps those patients with defective DNA repair pathways. On the basis of our findings on the DNA-repair signature, we estimate that only one of nine patients with triple-negative disease may have defective DNA repair. However, we acknowledge that this signature was derived from triple-negative breast tumors, and the results and interpretation are hypothesis-generating and should be considered with caution.

## Conclusion

To our knowledge this is the first report that stem cells are enriched in the normal adjacent breast tissue of patients with triple-negative breast cancer. The presence of stem cells may be an important predictive marker for the risk of developing triple-negative breast cancer. However, temporality and functional studies are needed to determine a causal relationship between stem cells and the incidence of breast cancer. Establishing the etiologic relationships between tumor subtypes by using normal epithelial subsets has profound implications for the development of clinically useful diagnostic and prognostic markers, as well as targeted therapies.

## Abbreviations

ALDH1: Aldehyde dehydrogenase; BRCA1: Breast cancer 1 gene; DAPI: 4',6-diamidino-2-phenylindole; ER: Estrogen receptor; GADPH: Glyceraldehyde-3-phosphate dehydrogenase; Her2: Human epidermal growth factor-2; PARP: Poly(adenosine diphosphate-ribose) polymerase; PM/MM: Perfect match/mismatch; PR: Progesterone receptor.

## Competing interests

The authors of this manuscript have no competing interests.

## Authors’ contributions

RLA participated in conception and design, collection and assembly of data, data analysis and interpretation, financial support, and manuscript writing. WTY and DGR carried out collection and assembly of data. MDL and HW participated in collection and assembly of data. MTL participated in data analysis and interpretation, and financial support. CJC carried out data analysis and interpretation. KRS and SGH participated in data analysis and interpretation. AAS participated in collection and assembly of data, data analysis and interpretation, and financial support. AMB participated in data analysis and interpretation, and manuscript writing. WAW participated in interpretation, conception and design, and manuscript writing. JCC participated in conception and design, and financial support. All authors read and approved the final manuscript.

## Supplementary Material

Additional file 1: Figure S1 Stem-cell cell surface markers were present in the triple-negative normal adjacent tissues to a much greater extent than in the estrogen receptor-positive (ER^+^) sample. (A) Normal adjacent tissue from patients with triple-negative cancer; arrows indicate areas positive for CD44^+^/CD49f^+^/CD133/2^+^(40×). (B) Normal adjacent tissue from patients with ER^+^ disease. Scale bar 50 μm; red, CD4; purple, CD49f; green, CD133/2; blue, 4',6-diamidino-2-phenylindole (DAPI), and combined CD44^+^/CD49f^+^/CD133/2^+^. Click here for file
