# Supplementary figures and images for: Cancer stem cell markers are enriched in normal tissue adjacent to triple negative breast cancer and inversely correlated with DNA repair deficiency
Source: Breast Cancer Res. 2013 Sep 4;15(5):R77. doi: 10.1186/bcr3471 (PMC4053576; doi:10.1186/bcr3471)

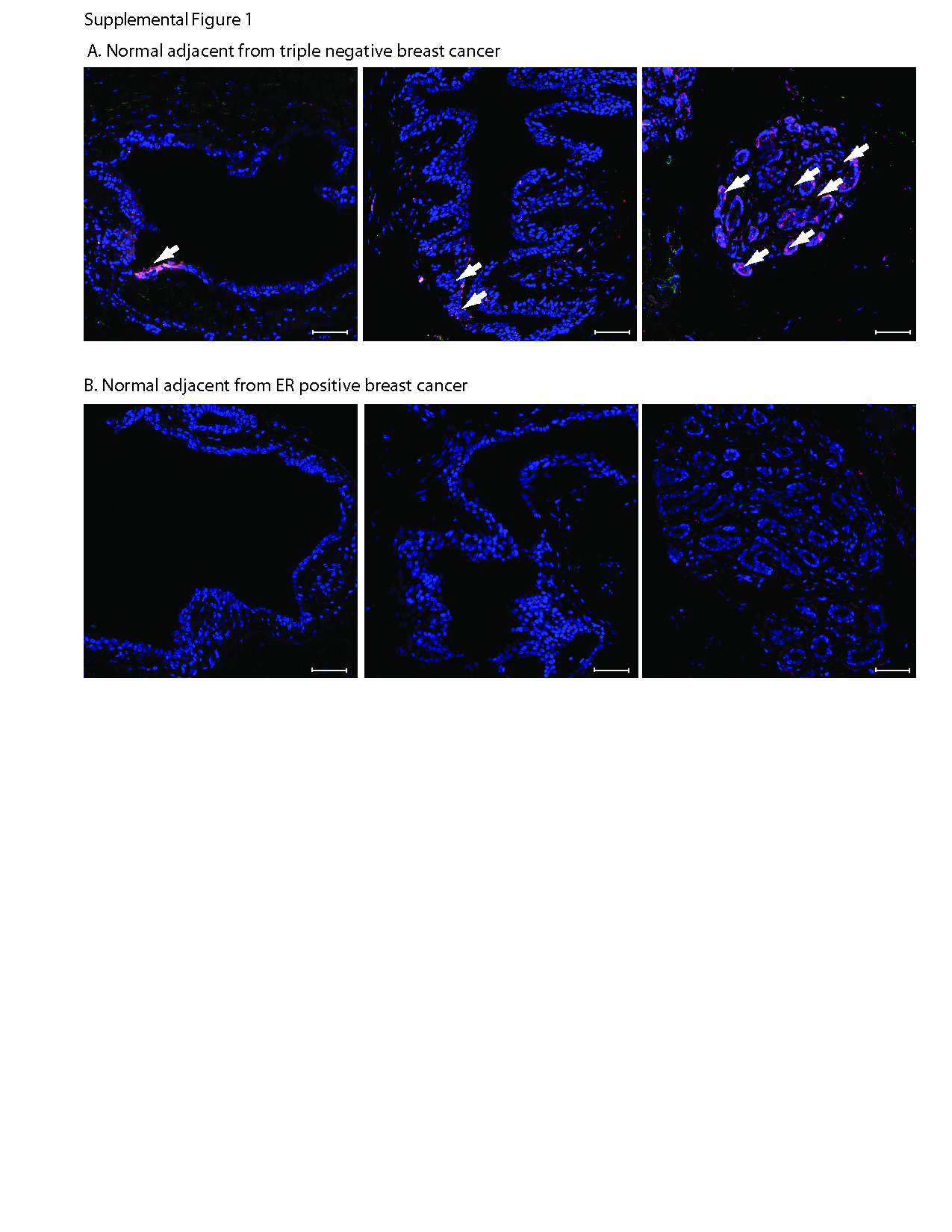

Supplement: Additional file 1: Figure S1 — Stem-cell cell surface markers were present in the triple-negative normal adjacent tissues to a much greater extent than in the estrogen receptor-positive (ER+) sample. (A) Normal adjacent tissue from patients with triple-negative cancer; arrows indicate areas positive for CD44+/CD49f+/CD133/2+(40×). (B) Normal adjacent tissue from patients with ER+ disease. Scale bar 50 μm; red, CD4; purple, CD49f; green, CD133/2; blue, 4',6-diamidino-2-phenylindole (DAPI), and combined CD44+/CD49f+/CD133/2+. [file bcr3471-S1.tiff]
